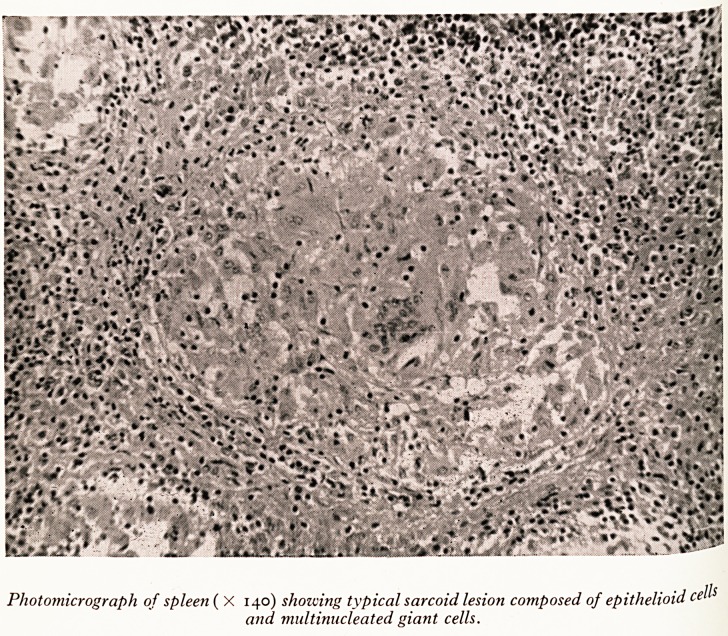# Sarcoidosis Treated with Cortisone

**Published:** 1957-07

**Authors:** T. F. Hewer


### SARCOIDOSIS TREATED WITH CORTISONE

*Clinical Pathological Conference held at Canynge Hall on 5th February 1957*

CHAIRMAN: PROFESSOR T. F. HEWER

*Dr. J. M. Naish:* This is a man of 37 who, at the age of 9, developed epilepsy. He had regular epileptic attacks between the ages of 9 and 23. I am not sure precisely what treatment he had during that time but after the attacks stopped at the age of 23 he went on having 1 gr. phenobarbitone at night and also a bromide mixture.

He first came under close medical scrutiny in the early part of 1954 when he was seen at the Central Health Clinic complaining of cough, loss of weight, scanty amount of sputum and some fever.

An X-ray of his chest showed a high right dome of the diaphragm and a rather large, glandular shadow in the right hilum. The E.S.R. was normal. There was no fever. The conclusion reached was that he was not suffering from tuberculosis but from some form of fibrosis of the lungs associated with bronchitis. Penicillin was recommended for treatment at home.

The next incident in this man's life was in September 1954, approximately nine months after the above X-ray was taken, when he was noticed to have rather a large "tummy" and complained of some abdominal pain. The following month he had rather more severe abdominal pain. His doctor sent him to Southmead Hospital to see Mr. Pocock, and he was investigated. His liver was enlarged and also his spleen. A barium meal showed no definite evidence of peptic ulcer, and liver function tests which were done at that time, in October 1954, showed the plasma globulin to be very much raised, being 5.5 g. per cent while the albumen was 3 g. per cent., E.S.R. 13 mm./hr. I think no definite conclusion was reached at this time as to the cause of the hepatosplenomegaly and he was discharged from hospital, the pain having settled down.

He was readmitted in January 1955, two and a half months later, with acute abdominal pain. An anterior perforated gastric ulcer was found at laparotomy. It was sewn up and he made quite a good recovery. He was discharged from Southmead to the recovery ward for surgical cases at Snowdon Road Hospital and while he was there his general condition was rather poor and he was about 2 stone under optimum weight so they asked me to see him. I was very interested by this combination of fibrosis in the lungs with enlarged liver and spleen, so we took him over to Southmead and did a liver biopsy with a "Terry" needle. This showed the typical appearances of Boeck's sarcoidosis, which was the diagnosis clinically suspected because of the rather peculiar combination of lung fibrosis, splenomegaly and hepatomegaly. The patient was reasonably well in himself. There was no fever and no change in peripheral blood count. Hb was about 85 per cent.

Acting on the assumption that sarcoidosis may be a reaction to tuberculous infection with massive production of fibrous tissue, this patient was treated with streptomycin, P.A.S. and cortisone. That treatment is followed, I should say, by about half the physicians in this country for patients with sarcoidosis who have substantial symptoms. We felt that with this man, who was underweight with a grossly enlarged liver and moderately enlarged spleen, it was justified. We kept him on this treatment for nearly a year, and watched him in Out-Patients.

In March 1955, seven months after the start of treatment, he had rather nasty paronychiae of his toes, oozing thin pus, so I decided to wean him off cortisone in view of the probability that cortisone was interfering with his response to infection.

We then readmitted him in January 1956. By this time he was off his cortisone and we consulted the surgeons who removed several of his toe-nails and the infection cleared up. When all had healed we put him back on streptomycin, P.A.S., I.N.H. and cortisone 100 mg. per day. During the subsequent half-year, to July 1956, he improved very much, gained nearly 2 stone in weight and his liver and spleen decreased very much in size, the liver being barely felt. Cortisone was reduced to 75 mg. per day in May 1956 and to 50 mg. in October 1956.

During the whole of this time he was having a small dose of phenobarbitone because of this old history of epilepsy. He got back to work—he was a grocery manager—and he worked during the whole of the latter part of 1956. He had no trouble with epilepsy during the whole of this time. Then, on 21st November 1956, he started, quite suddenly, having numerous epileptic attacks. I am not sure of the precise treatment given by his own doctor but he was sent to hospital the following day and found to be dead on arrival—he died in the ambulance.

Here is a man, epileptic in childhood and early youth, who developed Boeck's sarcoidosis at the age of 36. This was treated with anti-tuberculous drugs and cortisone, which had good general effect with reduction in the size of liver and spleen. He died in status epilepticus after having had no epilepsy for fifteen years.

*Professor C. Bruce Perry:* Was there any change in the radiological picture of the chest over the eighteen months or so?

*Dr. Naish:* I think there was some clearance there.

*Professor Perry:* Did you think the shadow to the right of the heart was due to enlarged glands?

*Dr. Naish:* I thought there were some enlarged glands but also some partial collapse of a basal segment of the lower lobe.

*Professor Perry:* Is it true that he was given hydantoin for his epilepsy? It says so on the sheet.

*Dr. Naish:* I don't remember that he was and I don't know where that statement came from.

*Dr. N. J. Brown:* It says so in the case notes.

*Professor Perry:* So he did have fits which were treated?

*Dr. Brown:* He may have been put on it to prevent fits. It states in the notes that he was treated with it.

*Dr. Naish:* It certainly wasn't prescribed by us! We were never consulted about the epilepsy—he had not had any fits in all these fifteen years.

*Dr. Brown:* It may be that he had been put on it as a prophylactic measure before he came in and it was continued in hospital.

*Professor Hewer:* Dr. Mather has had a lot of experience in, and has made a special study of sarcoid. Perhaps he would like to comment on this case.

*Dr. H. G. Mather:* Yes. I think it is very unusual in as much as he had an enlarged liver as well as enlarged spleen. An enlarged liver in this disease is unusual. In a series of 93 cases no liver enlargement was demonstrated, although 14 per cent. had enlarged spleens. It has now become generally realized that many different manifestations of this condition are, in fact, all one disease. They used to be given various different names according to whether they were skin lesions or whether they were in the eye—uveitis—or whether they were in the bones. We now know that it is all one disease—sarcoidosis. A very important manifestation is uveitis and this is one of the reasons for making a firm diagnosis because it can be relieved by cortisone. We now know that the various skin manifestations are comparatively rare. Bone manifestations are even rarer and merely form part of the overall disease. We don't know anything about the cause. With regard to treatment, I entirely agree with Dr. Naish—we should give cortisone.

*Professor Hewer:* You say uveitis can be cured by cortisone?

*Dr. Mather:* Yes. I think everyone with severe uveitis should have cortisone. I have seen several people go blind and this can be prevented by cortisone. This patient had no changes in his eyes.

*Question:* Is it a killing disease?

PLATE IX

*Spleen, showing the very numerous sarcoid nodules.*

PLATE X

*Surface of lung showing sub-pleural sarcoid lesions* ($\times$ 1$\frac{1}{2}$).

*Photomicrograph of spleen (× 140) showing typical sarcoid lesion composed of epithelioid cells and multinucleated giant cells.*

*Dr. Mather:* Very rarely. Many cases are asymptomatic and the bulk of people suffering from sarcoidosis are far best left alone; 63 per cent clear within a year without any treatment. Leave well alone unless there are symptoms and the most important symptom is uveitis.

*Professor Hewer:* Unless uveitis or other symptoms occur, you would prefer to do nothing?

*Dr. Mather:* Yes. It is a killing disease in only a small percentage of cases. We aim to prevent progressive fibrosis in the lungs.

*Professor Perry:* The tragedy is once fibrosis starts, cortisone does not help.

*Dr. Mather:* Quite true.

*Professor Perry:* Did any of your cases present with erythema nodosum?

*Dr. Mather:* Yes; 12 per cent.

*Dr. N. J. Brown (Presenting the autopsy findings):* There is no mystery about this case. The diagnosis has already been given you and it is not going to be changed. The importance of this case is that it gives us an opportunity to study the lesions of Boeck's sarcoid at a stage when you don't usually see them. They don't die of it in this stage and if they die of sarcoid you only see " burnt out " lesions.

This man died of asphyxia due to status epilepticus, as a result of continued spasm of the muscles of respiration and possibly also of the larynx. By dying in this manner he afforded us a most rare opportunity of studying the lesions of another disease—sarcoid—from which he happened also to be suffering.

First of all a few words about the disease. It was first described as a skin disease by Jonathan Hutchison in 1869. It was first called sarcoid by Boeck in 1899 and he also described the histological appearances. Boeck was a Norwegian physician who died in 1917. The disease is an inflammatory one in which one finds granulomatous lesions of a characteristic type in various parts of the body. They all used to have different names; if they were in the skin, they were called Boeck's sarcoid; if in the eye—Heerfordt's disease or uveo-parotid fever; if in the bones—osteitis tuberculosa multiplex cystica, and so on. It is now realized that these different manifestations are all part of the same disease—sarcoidosis. Generalized sarcoidosis, as in this case, is sometimes called Schaumann's disease. Schaumann was a Swedish dermatologist who described the generalized type in 1914. You will notice the number of Scandinavian names associated with this disease; this is a reflection of the fact that the disease is commoner in Scandinavia than anywhere else in the world.

This case is a beautiful example of generalized sarcoidosis with lesions in many parts of the body. I shall show you a large number of pictures and describe the lesions to you as we go along.

*Dr. Brown then showed lantern slides and photomicrographs of the various organs.*

In the spleen (Plate IX), there were large numbers of whitish, nodular lesions each 1–2 cm. in diameter, spread evenly through the substance of the organ and appearing beneath the capsule. The spleen was greatly enlarged, being approximately six times the normal size.

In the liver, there were numerous similar lesions but the individual nodules were rather smaller.

There were enlarged lymph nodes all over the body, the largest one being approximately 2 cm. in diameter in the para-aortic region. For the most part they were discrete but in places they were matted together. Some showed a uniformly pink cut surface but in some there were pale miliary nodules.

The lungs showed whitish-pink, nodular lesions beneath the pleura (Plate X) but these were not easily seen on the cut surface although it was possible to palpate large numbers of nodular lesions throughout the substance of both lungs.

Microscopically, the lesions all had a similar appearance. The nodules were made up of aggregations of sarcoid follicles composed of large multinucleated giant cells, some epithelioid cells with a rather reticulated cytoplasm, and a surrounding zone of lymphocytic infiltration (Plate XI). The follicles had a clearly defined outline and showed no caseation. No acid-fast bacilli were demonstrated in them. Many of the giant cells were particularly large and striking, one, in the lung, having as many as 57 nuclei. There was little surrounding fibrosis. The lesions differ from miliary tubercles in that there is no caseation, no acid-fast bacilli are seen, the giant cells are larger and the lesions are more discrete. Reticulin impregnation showed a fine reticulin pattern throughout the lesions, while in tuberculosis the reticulin pattern is usually destroyed. In the lung, sarcoid follicles were present in the walls of the bronchioles and were particularly frequent in the walls of small arteries, in some of which they infiltrated into the lumen with resulting thrombosis. In the lymph-nodes, there were large masses of homogeneous eosinophilic material. This superficially resembles amyloid but does not take the characteristic pink stain with gentian violet. It is known as paramyloid and is a characteristic finding in sarcoidosis. A few lesions were demonstrated microscopically in the kidney but the largest number were found in liver, spleen, lung and lymph nodes. No sarcoid lesions were demonstrated in the brain or meninges.

The lesions were very typical of generalized sarcoidosis. Sometimes, in the giant cells, you see peculiar inclusions known as asteroid bodies. These were not easy to find in this case and unfortunately photographs were not available.

In conclusion, one might say a little about the theories of aetiology of this condition. Sarcoidosis has been variously considered to be:

(1) A chronic granuloma resembling tuberculosis but caused by some as yet undiscovered agent;
(2) An atypical form of tuberculosis;
(3) An atypical response on the part of the reticulo-endothelial system to tuberculosis or a form of hypersensitivity reaction.

*Professor Hewer:* One of the reasons for linking this disease in one's mind with tuberculosis is that the tuberculin reaction is usually firmly negative. Is that not so?

*Dr. Mather:* No. That is not true; 44 per cent. are negative to tuberculin 1 : 100 dilution. The diagnosis of sarcoidosis cannot be ruled out by a positive tuberculin test.

*Dr. Naish:* Did some of your cases convert from negative to positive?

*Dr. Mather:* No.

*Dr. J. E. Cates:* This is one of the most important cases we have had here for a long time. We have had a man on P.A.S., streptomycin and cortisone for a long time and he has accidentally died. I wonder whether you would now say that cortisone and other things have had any effect on him at all. This ought to help us decide whether such treatment will help us with our future cases.

*Dr. Naish:* I was merely following the example of authorities on the subject who had—at the time when this man first came under observation—noticed considerable improvement in the radiological appearance of the lung lesions after this treatment. And with this man there is no doubt that his liver decreased in size very considerably while under treatment but that may perfectly well have been the natural course of events. I would be the last to say treatment had any effect at all, but one believed it was honestly the best thing to do for the chap. I don't know whether one can argue an awful lot from the fact that his sarcoid lesions are still there.

*Dr. Mather:* I quite agree. One must not be afraid to treat sarcoid with potentially dangerous drugs. One treats many diseases without completely curing the lesions. We treat mitral stenosis, but there is still mitral stenosis at post-mortem. The treatment is to relieve symptoms. If they are got back to work it is a good job.

*Dr. O. C. Lloyd:* What happens if you treat sarcoidosis with cortisone without streptomycin and P.A.S. Do some develop tuberculosis if you give them that?

*Dr. Mather:* They may, but you may have been dealing with tubercle all along: hence it is important to make a firm diagnosis of sarcoidosis. People are afraid to give cortisone alone in case some die of tuberculosis, so we give P.A.S. and streptomycin as well as cortisone to prevent tuberculosis but many physicians now treat sarcoid with cortisone alone without any serious complication. There is a funny association between tuberculin sensitivity and sarcoid because if you took 100 normal people, you would expect less than 44 per cent. negative to 1 : 100 dilution. It is very difficult to make people with sarcoid tuberculin positive even by giving B.C.G. Cases of Hodgkin's disease have a negative Mantoux in a similar proportion to patients with sarcoidosis, suggesting that the anergy may be due to the reticulo-endothelial involvement.

## Figures and Tables

**Figure f1:**
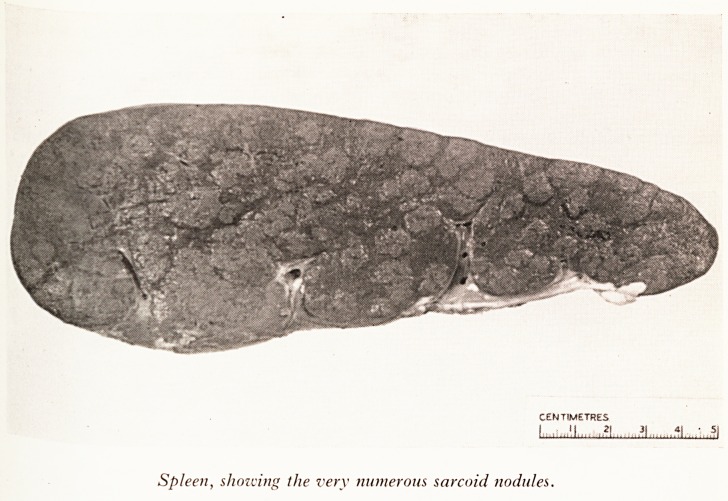


**Figure f2:**
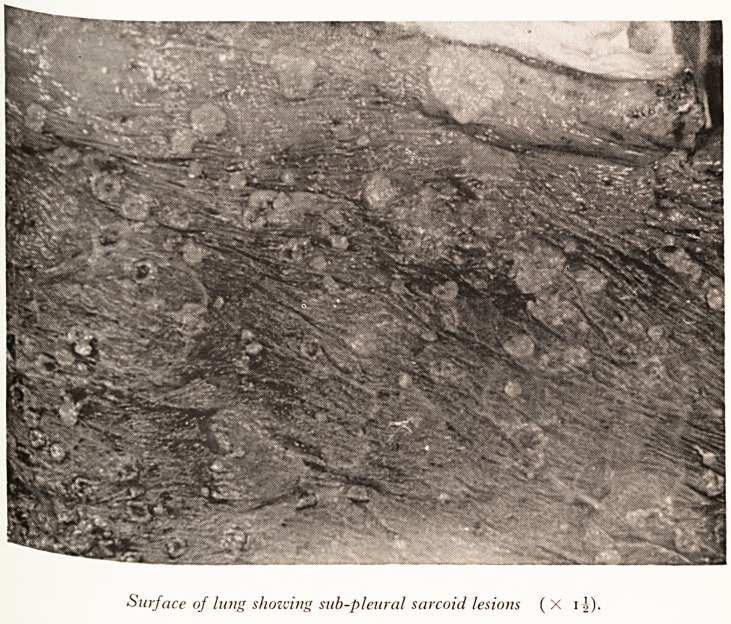


**Figure f3:**